# Research on the Influence of Information Diffusion on the Transmission of the Novel Coronavirus (COVID-19)

**DOI:** 10.3390/ijerph19116801

**Published:** 2022-06-02

**Authors:** Shanlang Lin, Chao Ma, Ruofei Lin

**Affiliations:** School of Economics and Management, Tongji University, Tongji Building A, Siping Road 1500, Shanghai 200092, China; linshanlang@163.com (S.L.); linruofei2021@163.com (R.L.)

**Keywords:** COVID-19, information diffusion, disease transmission

## Abstract

With the rapid development of the Mobile Internet in China, epidemic information is real-time and holographic, and the role of information diffusion in epidemic control is increasingly prominent. At the same time, the publicity of all kinds of big data also provides the possibility to explore the impact of media information diffusion on disease transmission. We explored the mechanism of the influence of information diffusion on the transmission of COVID-19, developed a model of the interaction between information diffusion and disease transmission based on the Susceptible–Infected–Recovered (SIR) model, and conducted an empirical test by using econometric methods. The benchmark result showed that there was a significant negative correlation between the information diffusion and the transmission of COVID-19. The result of robust test showed that the diffusion of both epidemic information and protection information hindered the further transmission of the epidemic. Heterogeneity test results showed that the effect of epidemic information on the suppression of COVID-19 is more significant in cities with weak epidemic control capabilities and higher Internet development levels.

## 1. Introduction

In December 2019, a number of cases of viral pneumonia with unknown causes were found in Wuhan, which were confirmed as the novel coronavirus 2019 (COVID-19) [1]. Despite strict interventions such as isolation treatment and traffic control, the epidemic spread rapidly to all provinces and cities in the country at an unprecedented rate. As of 25 February 2020, according to the reports of 31 provinces (autonomous regions, Municipality) and Xinjiang Production and Construction Corps, a total of 77,271 confirmed cases, 3434 suspected cases, 2596 deaths, and 25,065 cured cases have been reported [2]. This is another major public health emergency in China after the attack of the SARS virus in 2003.

Since the outbreak of COVID-19 occurred around the Spring Festival, the scale of population movement was large and the frequency was high, which also increased the difficulty of epidemic prevention and control [3]. In order to prevent the spread of the epidemic, the central government has taken unprecedented prevention and control measures, including setting up designated admission hospitals, expanding the supply of beds in the hospital, coordinating the dispatch of medical prevention and control materials, extending the Spring Festival holiday, implementing peak staggering return, measuring the temperature of vehicles and stations, disinfecting, ventilating, etc. The National Health Commission also sent a number of supervision teams to hospitals and disease control agencies to conduct on-site supervision. Wuhan also announced the closure of the city on 23 January, suspending urban public transport and strictly controlling the access of people inside and outside Wuhan [4]. Subsequently, 31 provinces, regions, and cities in the country successively launched the level 1 emergency response to public health emergencies, strictly controlled the transmission of the virus, and made every effort to prevent the further spread of the epidemic [5].

Since SARS in 2003, China has implemented legislation on the surveillance, reporting, and early warning system of infectious diseases, requiring the regular release of information during public health emergencies [6]. There is a clearly defined procedure and schedule for reporting public health emergencies which requires designated medical centers to submit relevant information online. If confirmed, reports on SARS and other infectious diseases can be submitted and received directly within two hours through the Internet [7]. Although less information is available in the early stages of the COVID-19 outbreak, there is an understandable delay in identifying a small number of severe respiratory cases [8]. After 20 January 2020, with the outbreak of the epidemic and the release of information, COVID-19 epidemic information became the most concerned information of the public, and media reports entered a white-hot stage. The emergence of the COVID-19 pneumonia has also caused widespread panic among the public. The official media, micro-blog, WeChat and other media have followed up the reports of real-time epidemic, new symptoms, and prevention measures, and conveyed clear and positive information in a timely fashion to society and advised the public to protect themselves and to view the epidemic more objectively [9,10]. Will the information diffusion help to eliminate rumors and guide the public to do a good job in protection and further inhibit the spread of the epidemic? Therefore, understanding the impact of information diffusion on epidemic transmission can help improve the prediction of epidemics and find preventive measures to slow down the spread of diseases.

Therefore, this paper studied the problems abovementioned. The innovation points of the research are as follows: First, summarizing the epidemic theory; through complex network analysis and analysis of the temporal and spatial background of the COVID-19 spread, we expounded the mechanism of epidemic transmission; second, we used the econometric method to conduct a regress, and acquire the basic conclusion that information diffusion can effectively reduce the spread of COVID-19; third, using big data mining technology, Baidu search index, and Baidu population migration, prevention, and control data during the epidemic were mined through Baidu and government information websites at all levels.

The remaining sections of the paper are as follows: the second section is the literature review and mechanism analysis; the third section is the methodology, which describes the model construction, data source, main variable calculation, and statistical description; the fourth section is empirical analysis; and the fifth section is the conclusions and implications.

## 2. Literature Review and Mechanism Analysis

### 2.1. Literature Review

Since the beginning of the 21st century, due to the outbreak of SARS, avian influenza, novel H1N1 influenza, and Ebola cross the world, the public has been increasingly concerned about emerging infectious diseases, and the problem of disease transmission has been widely studied [11]. In general, the spread of an epidemic is considered to be a dynamic process in which the disease passes from one individual to another through contact between individuals on the contact network [12]. Disease transmission often occurs in a dynamic social environment, and individual health behavior decision-making is guided by cultural norms, peer behavior, and media reports [13]. Although vaccination is a major strategy to protect individuals from infection, the development, testing, and production of new vaccines often take a long time [14]. Before receiving enough vaccines, the best protection for individuals is to take preventive actions, such as wearing masks, washing hands frequently, taking drugs, avoiding contact with patients, etc. (Centers for Disease Control and prevention, 2008). The historical experience of SARS tells us that effective control measures, such as early identification and isolation of SARS cases, tracking and isolation of the contacts, travel restrictions, and raising public awareness of risk, can help to contain the spread of the virus [15].

As the public gradually realized the importance of personal behavior in preventing the spread of infection, researchers began to explore the mathematical model of disease transmission including personal behavior. These models have been used to guide strategies for disease transmission control [16] and quantify the role of individual protective measures in controlling several outbreaks, including the Ebola virus outbreak in West Africa in 2014 [17], the SARS outbreak in Hong Kong in 2003 [18], and the H1N1 outbreak in central Mexico in 2009 [19]. Some scholars also assessed the epidemic trend and studied the progress of the epidemic in different parts of China based on the public epidemiological data of COVID-19 [20].

Understanding the impact of the media on the spread of the disease can help improve the prediction of epidemics and identify preventive measures to slow the spread of the disease. Social media platforms have been used as information channels for citizens, and their importance has been further recognized due to the lockdown policies implemented by governments to curb the spread of COVID-19 [21]. However, there is a lack of direct verification of the containment of the epidemic, especially from econometric methods. The widespread misuse of social media—leading to the dissemination of false, alarmist, and exaggerated information—has received more attention [22,23]. However, due to information control in China, and internet regulation by the Chinese government, misleading information is often deleted quickly, and the negative impact is reduced to a low level. Many models also link the disease-related media transmission with the protection function, usually assuming that the influence of media will reduce the effective transmission rate and slow down the spread of diseases. These studies indicate that the impact of media increases with the number of people infected [24], or both with the number and the rate of change [25,26]. When the number of cases is high or the prevalence of diseases increases rapidly, the information diffusion slows down the spread of diseases and creates interesting disease transmission dynamics, such as multi-wave outbreaks [27]. However, it is not clear whether the media function formalization proposed by the model fully reflects the actual influence. The choice of media function directly affects the form of disease transmission, making the accurate parameterization of the media the key [28].

However, most of the current research only focuses on the development of the disease itself on the complex network, as well as the impact of protective measures on the spread of the disease. There are relatively few studies on the spread of disease-related information, and only a few of them are carried out through numerical simulation, with the preconditions being too idealized and too dependent on the setting of parameters. In addition, the model has just begun to consider how to combine the data from actual media reports [29], lacking the econometric analysis based on real-time data. In decade years, China’s Internet has experienced unprecedented development; various online social media based on the Internet (such as major search engines, social networking sites, news sites, etc.) have been integrated into people’s daily life, providing a broad platform for the dissemination of all kinds of information. Compared with the outbreak of SARS in 2003, the economic links between regions are increasingly close. Especially during the Spring Festival in China, the population flow is more frequent. The government has taken unprecedented measures to prevent and control the epidemic, and the official media and social media have released the latest epidemic information in a timely fashion [30]. By summarizing the related literature, we propose the hypothesis that information diffusion is helpful to curb the transmission of novel coronavirus.

### 2.2. Analysis of Mechanism

**Figure 1:**
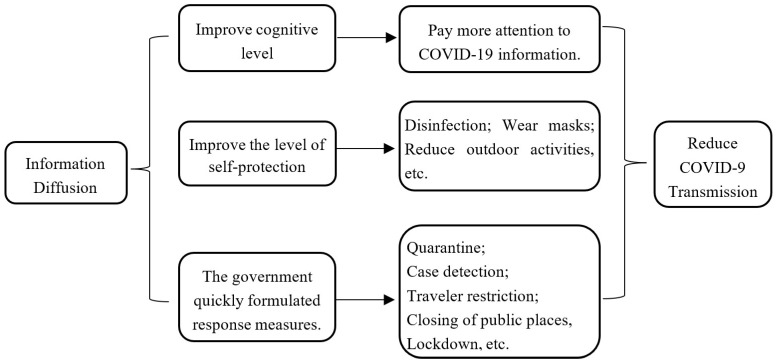
Analysis of the mechanism of information diffusion to reduce COVID-19 transmission.

Every outbreak of infectious disease in history will be filled with all kinds of information, and human cognition is constantly refreshed in the spread of information. Except for the timely release of news from the official media, social media played a crucial role in the spread of the epidemic information. If a city that adopts strict control measures, newspapers have to temporarily suspend their publications, and more people use social media and news apps to keep abreast of the latest developments. The dissemination of information related to infectious diseases will promote us to learn and accept new knowledge, increase the channels for people to perceive risks, and enable them to make health-friendly decisions [31]. In addition, wrong information will bring more discomfort, confuse the public’s normal perception and rational judgment, and cause a public trust crisis and collective panic. At the beginning of this epidemic, the World Health Organization believed that social media aggravated public concerns [32]. In general, accurate information dissemination will improve people’s cognition level, strongly influence people’s behavior, and change the effectiveness of government response measures (Figure 1) [33,34]. Therefore, we propose Hypothesis 1: epidemic information diffusion inhibits the transmission of COVID-19.

(1) Improve cognitive level. Different from the one-way and linear transmission mode of traditional media, the Internet has the characteristics of real-time and two-way interaction in the transmission of information, and has higher transmission efficiency [35]. Different from other information on the Internet, health information is highly sensitive information [36]. Users usually participate in the information diffusion process for the purpose of solving health problems after perceiving the risk of disease. When an infectious disease spreads among people, information about the disease will also spread immediately. Historical experience shows that information transmission situation is positively correlated with disease infectivity. In the early stage of COVID-19, a small amount of information about the novel coronavirus was spread, mainly focusing on the popular science of the virus and the current infection dynamics. These messages began to attract the attention of the public, and many people chose to continue to pay attention to the information. When the full-blown outbreak occurred, they were less likely to be infected because they had a more comprehensive understanding of the epidemic and were quicker to respond. Due to the high infectivity and transmission ability of novel coronavirus, the public pays special attention to the information of novel coronavirus. They are all eager to have a comprehensive understanding of the transmission mechanism of novel coronavirus, so information diffusion has significantly improved the public’s cognition.

(2) Improve the level of self-protection. When COVID-19 enters the rapid outbreak period, epidemic information will be spread on major media. When individuals know the existence of the disease, they tend to change their behavior, such as wearing masks and becoming vaccinated, to avoid being infected by the virus. This change will have an impact on the spread of the disease. Susceptible (or infected) people who have information about COVID-19 will disconnect from those around them who are infected (or susceptible) to prevent further spread of the disease. For example, in the first hour after the 8.8-magnitude earthquake in Japan in 2011, there was a huge amount of tweets on Twitter, with Tokyo generating about 1200 Twitter messages per minute. These messages help families or individuals to convey first-hand information on the scene, and enable the related individuals to make timely preparations and protection [37]. In the prevention and control of the Ebola virus epidemic, Nigeria has a lower infection rate than neighboring Liberia. Many scholars believe that social media has played a role in the demonstration of behavior [38].

(3) The government quickly formulated response measures. Media reports and public sentiment can have a significant impact on the public and private sectors in deciding whether to stop certain services, including air services. After receiving the epidemic information, governments at all levels also promptly initiated emergency response plans, and gradually adopted a series of response measures, such as early detection and quarantine, travel restrictions, closing public places, and lockdown, which greatly reduced the further spread of novel coronavirus [39,40,41]. Numerous studies have shown that travel restrictions are a measure to contain the spread of novel coronavirus. For example, the travel restrictions in Wuhan delayed the overall progress of the epidemic in mainland China by 3–5 days, but the impact on the international scale is more significant [42]. The Italian government also imposed travel restrictions and cancelled all public events in the northern region [43].

**Figure 2:**
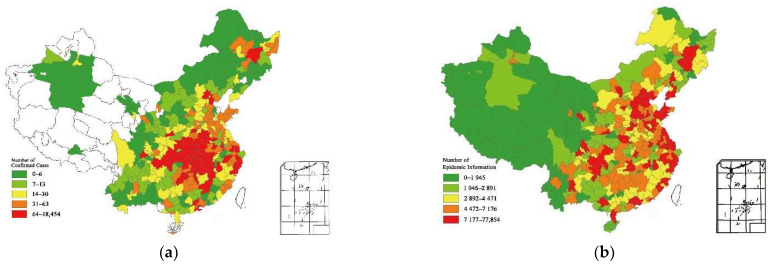
The geographical distribution of COVID-19 and epidemic information in China as of 10 February 2020. (**a**) The confirmed cases are distributed radially from Wuhan. (**b**) Geographical Distribution of Epidemic Information in China as of 10 February 2020.

In addition, we visualized the geographical distribution of COVID-19 and epidemic information (Figure 2). We can find that the cities with the most epidemic information are not the cities with the most severe epidemic, such as Hubei Province, but developed regions such as Beijing, Shanghai, and Guangdong Province. The epidemic situation in these regions is still serious, but relatively stable compared with Hubei Province. From the perspective of the disease transmission process in the past, the influence of epidemic information on the disease transmission is also affected by many other factors, such as the geographic location, the information level of the city, and the administrative efficiency of the local government. Therefore, it is necessary to further explore the epidemic transmission in different regions. We propose Hypothesis 2: under different epidemic control ability and information transmission efficiency, epidemic information presents different characteristics for the transmission of novel coronavirus.

## 3. Methodology

### 3.1. Model Construction

In general, infectious disease transmission is considered to be a dynamic process in which the disease spreads through contact between individuals on a contact network. Infectious disease transmission based on the Susceptible–Infected–Susceptible (SIS) model or the Susceptible–Infected–Recovered (SIR) model have been developed for decades. Many studies have focused on the process of disease transmission in complex networks and social networks [44,45]. However, few efforts have been made to integrate information diffusion with human behavior, considering these two interactive processes. In fact, when disease information is spread among the population, people will naturally take some preventive measures, which in turn restricts the transmission of the disease.

**Figure 3:**
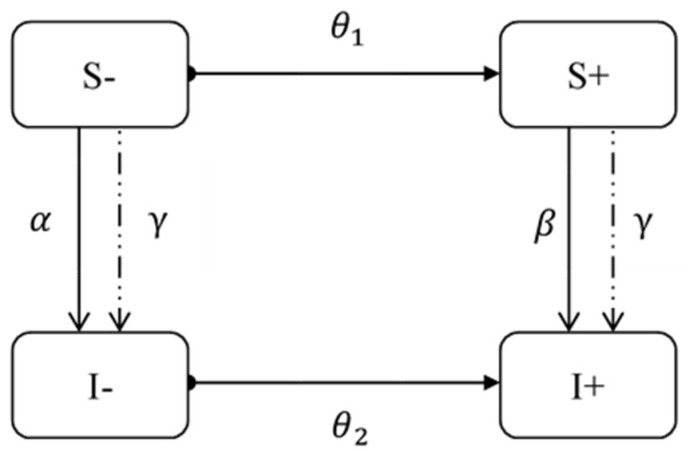
The process of information diffusion and COVID-19 transmission. The population is divided into four states: (1) S−: unconscious susceptible; (2) S+: conscious susceptible; (3) I−: unconscious infected; (4) I+: conscious infected. $\theta_{1}$, $\theta_{2}$, α and β denote the probability of state transition.

To explore the effect of information diffusion on epidemic outbreaks, we draw on the conceptual framework of Mao and Yang to develop a model of the interaction between epidemic and information [11]. We divided the population into three categories based on their health status: the susceptible (S), the infected (I), and the recovered (R). At present, it is not clear whether COVID-19 patients are re-infected after recovery (R), so we did not consider it here. In addition, we also divided people into two categories according to their level of information acquisition, namely conscious (+) and unconscious (−). Therefore, the population in the country can be divided into four states: (1) S−: unconscious susceptible; (2) S+: conscious susceptible; (3) I−: unconscious infected; (4) I+: conscious infected. As shown in Figure 3, we illustrate the process of information diffusion and COVID-19 transmission.

In the process of information diffusion, the Internet and other media reported epidemic-related information on a large scale. Some unconscious individuals in the susceptible group ($\theta_{1}$) received the information and became conscious individuals; and some of the unconscious individuals in the infected group ($\theta_{2}$) received the information and become conscious individuals.

In the process of COVID-19 transmission, unconsciously susceptible people (S−) are infected with probability α. Consciously susceptible people usually take self-protection measures such as wearing a mask, reducing going out, and disinfection, so they are infected with the probability of β (β<α). Due to isolation treatment for the infected patients, the infection probability of surrounding susceptible population (S+ and S−) are both γ.

Therefore, the information diffusion can reduce the transmission of COVID-19 in two ways. First, information diffusion will cause consciously susceptible people (β·S+) to take protective measures to prevent infection. Second, information diffusion will change unconsciously susceptible people ($\theta_{1}\cdot S-$) into consciously susceptible people, who then take measures to reduce infection ($\theta_{1}\cdot S-\cdot \beta$).

According to the hypothesis above, the empirical model of the influence of information diffusion on the spread of COVID-19 is as follows:

(1) $$XGBD_{ij}=\alpha +\beta_{1}search_{ij}+\beta_{2}X_{ij}+\xi_{i}+\xi_{j}+\varepsilon_{ij}$$

where, i denotes date; j denotes city; XGBD is the spread of COVID-19, which is measured by the number of cumulative and newly confirmed cases published by the National Health Commission each day; X is control variables, including traffic control (traf_con), social distancing (soci_dis), movement of population (migration), Population inflow rate of Wuhan (ratio), and GDP per capita (pegdp). $\xi_{i}$ is time fixed effect, $\xi_{j}$ is city fixed time, $\varepsilon_{ij}$ is the random error term.

### 3.2. Data Resource

To quantitatively explore the relationship between the transmission of COVID-19 and information diffusion, we first visited the Baidu Index website through Python to obtain the Baidu search index of the keywords related to the epidemic during the outbreak from 19 January to 10 February 2020 to measure the level of information diffusion. The data of infected cases during the corresponding period mainly came from the daily epidemic data released by the National Health Commission. The social distancing and traffic control data came from the public information of each city’s Health Commission website and government website on taking preventive and control measures, and they were scored uniformly according to the degree of control, and the corresponding values were added up. The national migration data and Wuhan’s outflow data came from Baidu Migration. The control variables at the city level came from China City Statistical Yearbook. In addition, cities without outbreaks were also excluded. After collation, 6417 observations from 301 cities were finally obtained.

### 3.3. Variable Description and Measures

Coronavirus transmission (XGBD): The number of confirmed cases is used to indicate the transmission of novel coronavirus in this paper. After the outbreak of the epidemic, the National Health Commission provided daily confirmed cases data. Therefore, we compiled a list of the daily number of cumulative confirmed cases (qzrs) and new confirmed cases (xzqz) in prefecture-level cities from 19 January to 10 February 2020.

**Figure 4:**
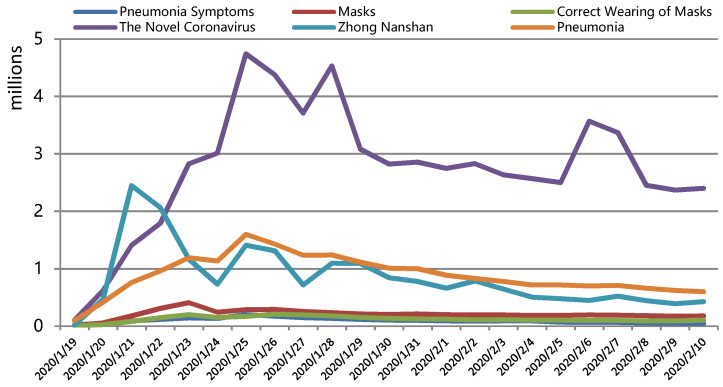
The number of six terms most relevant to the novel coronavirus epidemic searched by Chinese netizens.

Information diffusion (search): The main explanatory variable (information diffusion) in this paper was measured by the number of searches for epidemic-related information by the national people every day during the epidemic. Different from the media data used in previous studies as the level of information diffusion [28,46], the search index can better reflect people’s acceptance of information diffusion. Therefore, based on the search services provided by Baidu Index, six epidemic-related terms of “the novel coronavirus”, “pneumonia”, “Zhong Nanshan”, “pneumonia symptoms”, “masks”, and “correct wearing of masks” were selected as search terms, and the search index during the epidemic period (19 January–10 February) was crawled by Python, and the daily level of information diffusion of prefecture-level cities was summed up, as shown in Figure 4. In addition, the search index was divided into two categories: one is about the information on epidemic with “the novel coronavirus, pneumonia, Zhong Nanshan” (search1), and the other is about the protection with “pneumonia symptoms, correct wearing of masks and masks” (search2).

Traffic control (traf_con) and Social distancing (soci_dis): This paper collected and summarized the epidemic prevention and control mechanisms published by the Emergency Command of the COVID-19 Pneumonia Prevention and Control in each province and city, mainly including traffic control and social distancing [47]. According to these preventive and control measures taken by all prefectural administrative regions in the country, they were classified and scored into 15 items (see Table 1), each with a score of 1, starting from the time when each measure is implemented until the measure is cancelled.

For example, Shanghai began to implement the “isolation of close contacts of confirmed patients” for 14 days on 21 January. Since this measure belongs to “social isolation”, the “social isolation” score of Shanghai was 1 from 21 January. On 24 January, Shanghai began to implement the “partial cessation of public places in the city”, then the “social isolation” was added 1 point from 24 January, and so on. Finally, traffic control was carried out separately. The scores of each measure of social alienation were summed up, as shown in Table 2.

Population Flow (migration): As the epidemic occurred during the Spring Festival Movement in China, the large-scale population flow provided favorable conditions for the spread of the virus, and reasonable control of population flow helped to slow down the spread of the epidemic. Baidu Migration Big Data provides a migration index that reflects the scale of population migration into or out, and is comparable between cities. Therefore, the migration indexes of population moving in and out of prefecture-level cities were obtained respectively, and the indicators reflecting the overall population flow status of the city were summed up.

Other control variables. (1) The influx of population in Wuhan (ratio). The novel coronavirus epidemic first broke out in Wuhan, and the influx of Wuhan population may lead to the cross-city transmission of the epidemic. Baidu Migration big data provides the destinations and proportions of Wuhan’s daily population outflows. This paper selected the proportion of population flow in Wuhan to other cities to represent the population influx in Wuhan. (2) GDP per capita (pergdp). GDP per capita reflects the level of city social and economic development, while cities with high economic development tend to have more complete and stronger epidemic prevention facilities. The descriptive statistics of the main variables are shown in Table 3.

## 4. Empirical Analysis

### 4.1. Benchmark Regression

According to the econometric model constructed above, benchmark regression results were reported by controlling time and city fixed effects separately (see Table 4). Column (1) reports a direct regression on the two variables of information diffusion and COVID-19 transmission, and the results showed that the coefficient was significantly negative. Column (2) shows the result of adding traffic control and social distancing variables. Column (3) shows the results after adding further variables such as population flow, Wuhan inflow, and GDP per capita based on column (2). The regression results were also statistically significant and negative. This indicates that after controlling other factors affecting the COVID-19, information diffusion significantly reduced the transmission of COVID-19 in China. The coefficients of traffic control and social distancing were both significantly negative, indicating that local governments can help slow down the spread of the epidemic after implementing first-level response measures such as urban traffic control, isolation observation, and closed communities. In terms of variables reflecting population migration, the coefficient of urban population migration variable was significantly negative, indicating that the decrease in population inflows and outflows also reduced the spread of COVID-19; the coefficient of Wuhan population inflow variables was significantly positive, indicating that the population inflow in Wuhan has accelerated the spread of the virus to a certain extent.

From the first emergence of the COVID-19 to Wuhan lockdown after realizing the seriousness of the epidemic, the novel coronavirus spread widely in the city, which may affect the accuracy of the results. Therefore, we excluded Wuhan from the sample data and performed a regression test. As shown in column (4), the regression results were still stable and did not affect the significance of the coefficients.

### 4.2. Robustness Tests

#### 4.2.1. Robustness Test of Information Classification

To prove that the benchmark regression results are robust, we divide the information diffusion represented by the Baidu search index into two categories for further exploration. One is epidemic information (search1) that the public desires to know. The other is the self-protection information (search2) searched by the public to prevent themselves from being infected by the novel coronavirus.

The results are shown in Table 5. Columns (1) and (2) are the results of the robustness test, which show the impacts of epidemic information diffusion (search1) on the transmission of COVID-19; the regression coefficients were significantly negative. Columns (3) and (4) are the results of robustness test which show the impacts of the self-protection information diffusion (search2) on the transmission of COVID-19, and the coefficients were also significantly negative. It shows that both the epidemic information and self-protection information hindered the further transmission of COVID-19.

#### 4.2.2. Robustness Test of New Cases

The data released by the National Health and Construction Commission include the daily number of new cases, which can better reflect the spread of the epidemic every day. Therefore, we use the new confirmed cases instead of the cumulative confirmed cases in the previous model for robustness testing. The results are shown in Table 6. In column (2), the regression result using the comprehensive search index was significantly negative, which is consistent with the basic regression result. In column (3) and column (4), the regression results using epidemic information and self-protection information search index respectively were also significantly negative. This proves that the regression results are robust, that is, information diffusion reduces the spread of COVID-19.

### 4.3. Heterogeneity Test

The results of this paper may be affected by different epidemic prevention and control capabilities or information diffusion efficiency. Therefore, to explore the heterogeneous impact of information diffusion on COVID-19 transmission, we divided the cities in sample into three groups—high, medium, and low—based on epidemic prevention and control capabilities (GDP per capita) and information diffusion efficiency (Internet). Table 7 reports the results of urban epidemic prevention and control capacity. We can find that the coefficient of information diffusion was significantly negative in the medium and low epidemic control ability groups, and the coefficient value was larger in the low epidemic control ability group. This suggests that COVID-19 transmission could be suppressed better in a city with low epidemic prevention and control capacity. The results of Column (1) showed that the coefficient of information diffusion was not significant in cities with high epidemic prevention and control capacity. This may be due to the restrictive control measures and better medical conditions, which to the greatest extent limited the proliferation of COVID-19.

The level of Internet development in Chinese cities will also have an important impact on the diffusion of COVID-19 information. A higher level of Internet development means that the same amount of information can reach more people and have a higher efficiency of information diffusion. Therefore, we use the Internet Penetration Rate (Internet) of each province in the 39th National Internet Development Statistics released by China Internet Network Information Center (CNNIC) to measure the information diffusion efficiency, and divide the Internet penetration rate into high, medium, and low groups. As shown in Table 8, the regression results of information diffusion were significantly negative in columns (1) and columns (2). The value of the search variable is greater in column (1) than in column (2), indicating that COVID-19 transmission with high Internet development level was more effective. Therefore, it is of great significance for future epidemic prevention and control to accelerate the construction of network infrastructure in central and western regions and rural areas to improve the national Internet penetration rate.

## 5. Conclusions and Implications

We draw on the model of behavioral dynamics, use econometric methods and high-frequency data such as novel coronavirus epidemic data published by the National Health and Medical Commission, Baidu search index, and Baidu migration index to explore the relationship between information diffusion and the transmission of COVID-19. The results showed that: Firstly, after fixing year and city and controlling other variables that affect the spread of novel coronavirus, the information diffusion significantly reduces the transmission of COVID-19. After excluding Wuhan from the sample, the regression results were still robust. Secondly, two robustness tests showed that both the diffusion of epidemic information and self-protection information have significantly reduced the further transmission of COVID-19. This shows that timely and accurate information diffusion plays an important role in epidemic prevention and control after the outbreak. Thirdly, the current literature affirms the contribution of information diffusion, but ignores differences between cities. We found that in low-income areas with weak epidemic control ability, epidemic information had a more significant inhibition effect on the COVID-19. In addition, in cities with high Internet penetration, the suppression of COVID-19 by epidemic information was more significant.

Given the fact that the Internet is regulated by the Chinese government, most international news media and social networking sites are blocked in China [48,49], which may hinder the access of the public to information. However, Internet regulation can reduce public debate about the origins of the outbreak and keep focus on strengthening self-protection. Supervision can also prevent the excessive and inaccurate dissemination of epidemic information and reduce possible adverse effects. It is not clear whether Internet regulation will help or harm the spread of COVID-19, and further research is needed.

A number of implications can be drawn based on our findings: (1) The existence of Internet regulation can reduce the rumor information, but may also lower the information transparency. Therefore, the government convenes a press conference in a timely manner to disclose the epidemic situation information and make the information diffusion more transparent. In particular, the official media played a role of weathervane, and they need to follow up the epidemic report to let public know about the epidemic in a timely fashion. (2) Strengthen the supervision of information diffusion. Much of the literature has found widespread abuse of social media, which leads to the spread of false, alarmist, and exaggerated information, exacerbating health concerns. Official authorities, hospitals, and well-known experts need to quickly deny rumors for various purposes to avoid adverse social influences. (3) Different from developed countries, there is a gap in the Internet penetration rate between urban and rural areas in China. Therefore, governments at all levels need to issue official documents in a timely manner to transmit information to rural areas.

## Figures and Tables

**Table 1:** Items of traffic control and social distancing.

Traffic Control	Social Distancing
Launching level 1 response Suspending all the cross-city passenger transport Suspending part of the cross-city passenger transport Monitoring all the cross-city passenger transport Monitoring part of the cross-city passenger transport Suspending all the public transport Suspending part of the public transport	Closing all the public places Closing part the public places Closed management of all the community Closed management of part of the community Quarantining returnees from key epidemic area (Hubei) for 14 days Quarantining all the returnees for 14 days Quarantining the contact for 14 days Isolating and testing the suspected

Notes: Summary of measures taken in epidemic prevention and control from various cities. Traffic control mainly includes seven items, and social distance control mainly includes eight items.

**Table 2:** Traffic control and social distancing score of shanghai.

Date	Traffic Control	Social Distancing
19 January 2020	0	0
20 January 2020	0	0
21 January 2020	0	2
22 January 2020	1	2
23 January 2020	1	2
24 January 2020	2	4
25 January 2020	2	4
26 January 2020	3	4
27 January 2020	3	4
28 January 2020	3	4
29 January 2020	3	4
30 January 2020	3	4
31 January 2020	3	4
1 February 2020	3	4
2 February 2020	3	4
3 February 2020	3	4
4 February 2020	3	4
5 February 2020	3	6
6 February 2020	3	6
7 February 2020	3	6
8 February 2020	3	6
9 February 2020	3	6
10 February 2020	3	6

Note: It shows the daily scores of traffic control and social distancing control in Shanghai during the sample period studied in this paper.

**Table 3:** Statistical description of variables.

Variable	Description	Obs	Mean	Std	Min	Max
qzrs	The number of cumulative confirmed cases	6417	0.35	2.48	0	66.63
xzqz	The number of new confirmed cases	6417	0.05	0.47	0	28.63
search	Information Diffusion	6417	56.77	38.20	1.02	317.70
search1	Information Diffusion-epidemic	6417	49.65	34.18	0.81	294.20
search2	Information Diffusion-prevention	6417	7.11	4.67	0	54.57
traf_con	Traffic control	6417	2.42	1.63	0	4
soci_dis	Social distancing	6417	2.48	2.06	0	6
migration	population flow rate	6417	1.72	2.62	0.03	31.23
ratio	Population inflow rate of Wuhan	6417	0.31	1.74	0	23.86
pergdp	GDP per capita	6417	5.78	3.18	1.52	18.31

**Table 4:** Benchmark regression results.

	(1)	(2)	(3)	(4)
	Only Explanatory Variables	Gradually Add Control Variables		Exclude Wuhan City
	qzrs	qzrs	qzrs	qzrs
search	−0.0104 **	−0.00988 **	−0.00659 ***	−0.00603 ***
	(−2.26)	(−2.20)	(−3.48)	(−3.21)
traf_con		−0.142 ***	−0.0860 **	−0.0727 **
		(−2.78)	(−2.27)	(−1.97)
soci_dis		−0.113 ***	−0.0796 ***	−0.0592 ***
		(−4.72)	(−4.21)	(−3.32)
migration			−0.0458 ***	−0.0271 ***
			(−3.79)	(−3.05)
ratio			3.026 ***	3.027 ***
			(7.65)	(7.62)
pergdp			0.0709 *	0.0151
			(1.87)	(0.53)
Constant	0.00462	0.157	−1.035 ***	−0.585 *
	(0.02)	(0.70)	(−2.78)	(−1.89)
Fixed time	YES	YES	YES	YES
Fixed city	YES	YES	YES	YES
N	6417	6417	6417	6394
R^2^	0.5266	0.5302	0.6787	0.6870

Notes: Column (1) is the result containing only explanatory variables (search) and explained variables (qzrs). Column (2) and column (3) are the result of gradually adding control variables. Column (4) is the result after removing Wuhan from the sample. *t* statistics in parentheses, * *p* < 0.1, ** *p* < 0.05, *** *p* < 0.01.

**Table 5:** Robustness test of information classification.

	(1)	(2)	(3)	(4)
	Only Explanatory Variables	Add Control Variables	Only Explanatory Variables	Add Control Variables
	qzrs	qzrs	qzrs	qzrs
search1	−0.00873 *	−0.00567 ***		
	(−1.91)	(−2.90)		
search2			−0.184 ***	−0.112 ***
			(−3.40)	(−5.12)
traf_con		−0.0871 **		−0.0973 ***
		(−2.29)		(−2.61)
soci_dis		−0.0795 ***		−0.0832 ***
		(−4.21)		(−4.37)
migration		−0.0479 ***		−0.0329 ***
		(−3.94)		(−2.78)
ratio		3.031 ***		2.957 ***
		(7.64)		(7.66)
pergdp		0.0661 *		0.0311
		(1.74)		(0.89)
Constant	−0.0235	−0.959 ***	−0.446 ***	−0.952 ***
	(−0.12)	(−2.58)	(−3.20)	(−2.61)
Fixed time	YES	YES	YES	YES
Fixed city	YES	YES	YES	YES
N	6417	6417	6417	6417
R^2^	0.5254	0.6783	0.5393	0.6831

Notes: The regression results after we divide the information into two categories, search1 and search2. Columns (1) and (2) are the results of search1. Columns (3) and (4) are the results of search2. *t* statistics in parentheses, * *p* < 0.1, ** *p* < 0.05, *** *p* < 0.01.

**Table 6:** Robustness test of new cases.

	(1)	(2)	(3)	(4)
	Only Explanatory Variables	Add Control Variables	Replace the Explanatory Variable	
	xzqz	xzqz	xzqz	xzqz
search	−0.00115 *	−0.000665 **		
	(−1.72)	(−1.98)		
search1			−0.000632 *	
			(−1.76)	
search2				−0.00840 **
				(−2.55)
traf_con		−0.0108 *	−0.0108 *	−0.0119 *
		(−1.77)	(−1.77)	(−1.90)
soci_dis		0.00569	0.00570	0.00541
		(1.49)	(1.49)	(1.43)
migration		−0.00754 ***	−0.00769 ***	−0.00678 ***
		(−3.34)	(−3.40)	(−2.88)
ratio		0.381 ***	0.381 ***	0.376 ***
		(3.66)	(3.66)	(3.62)
pergdp		0.00328	0.000000326	−6.62 × 10^−8^
		(0.52)	(0.50)	(−0.10)
Constant	−0.0154	−0.0563	−0.0540	−0.0388
	(−0.50)	(−0.96)	(−0.88)	(−0.61)
Fixed time	YES	YES	YES	YES
Fixed city	YES	YES	YES	YES
N	6417	6417	6417	6417
R^2^	0.3934	0.4093	0.4092	0.4098

Notes: Robustness test using new confirmed cases (xzqz) instead of cumulative confirmed cases (qzrs). *t* statistics in parentheses, * *p* < 0.1, ** *p* < 0.05, *** *p* < 0.01.

**Table 7:** Heterogeneity test results of per capita GDP.

	(1)	(2)	(3)
	High Per Capita GDP	Medium Per Capita GDP	Low Per Capita GDP
	qzrs	qzrs	qzrs
search	−0.00124	−0.00370 **	−0.0137 ***
	(−1.38)	(−2.41)	(−3.28)
traf_con	−0.108 ***	−0.155 ***	−0.100
	(−2.92)	(−3.10)	(−1.42)
soci_dis	−0.0802 ***	−0.0522 ***	−0.0960 **
	(−3.63)	(−2.92)	(−2.24)
migration	−0.0209 **	0.0665 *	−0.313 ***
	(−2.18)	(1.91)	(−4.64)
ratio	0.934 ***	−0.799 *	4.188 ***
	(7.34)	(−1.96)	(9.39)
Constant	0.0624	−0.310 *	1.751 ***
	(0.40)	(−1.78)	(4.28)
Fixed time	YES	YES	YES
Fixed city	YES	YES	YES
N	2139	2139	2139
R^2^	0.5320	0.5481	0.7907

Notes: According to the difference in epidemic prevention and control capabilities (GDP per capita), the cities in the sample are divided into three groups: high, medium, and low. *t* statistics in parentheses, * *p* < 0.1, ** *p* < 0.05, *** *p* < 0.01.

**Table 8:** Heterogeneity test results of Internet penetration.

	(1)	(2)	(3)
	High Internet	Medium Internet	Low Internet
	qzrs	qzrs	qzrs
search	−0.0100 ***	−0.000694 ***	0.00106
	(−3.30)	(−2.70)	(1.58)
traffic_control	−0.127 **	−0.00346	0.0251 *
	(−2.16)	(−0.93)	(1.88)
soci_dis	−0.211 ***	0.0100 ***	−0.0325 ***
	(−5.66)	(4.19)	(−3.71)
migration	−0.0260 *	0.00817 ***	0.00847 *
	(−1.72)	(4.11)	(1.91)
ratio	3.090 ***	−0.776 ***	−0.00867
	(7.80)	(−12.97)	(−0.10)
pergdp	0.129 **	0.0191 ***	−0.000475
	(2.43)	(3.73)	(−0.08)
Constant	−1.967 ***	−0.109 ***	−0.0828 ***
	(−3.45)	(−4.02)	(−2.63)
Fixed time	YES	YES	YES
Fixed city	YES	YES	YES
N	3565	2668	184
R^2^	0.6882	0.7511	0.8208

Notes: According to the difference in information diffusion efficiency (Internet), the cities in the sample are divided into three groups: high, medium, and low. *t* statistics in parentheses, * *p* < 0.1, ** *p* < 0.05, *** *p* < 0.01.

## Data Availability

Data available on request due to restrictions, e.g., privacy or ethical.
